# Public knowledge and attitudes towards antibiotics and antimicrobial resistance (AMR) in vietnam: a cross-sectional study

**DOI:** 10.1017/ash.2025.10034

**Published:** 2025-07-31

**Authors:** Van Nhi Tran, Thuc Quyen Huynh, Pham Tuyet Nhi Nguyen, Thi Phuong Truc Nguyen, Hoang An Nguyen, Gregory Hurter, Si Tuan Nguyen, Minh Khoi Le, Minh Thong Le, Chan Khon Huynh, Phuong Thao Nguyen, Thi Thu Hoai Nguyen

**Affiliations:** 1 School of Biotechnology, International University, Vietnam National University of Ho Chi Minh City, Ho Chi Minh City, Vietnam; 2 Research Center for Infectious Diseases, International University, Vietnam National University of Ho Chi Minh City, Ho Chi Minh City, Vietnam; 3 Saint Michaels College, Colchester, VT, USA; 4 Thong Nhat Dong Nai General Hospital, Bien Hoa City, Dong Nai province, Vietnam; 5 University Medical Center, Ho Chi Minh City, Vietnam; 6 School of Biomedical Engineering, International University, Vietnam National University of Ho Chi Minh City, Ho Chi Minh City, Vietnam

## Abstract

**Background:**

Antimicrobial resistance (AMR) is a significant public health threat. Understanding public knowledge and attitudes toward antibiotic usage is essential for educational campaigns combating AMR. This study evaluates public knowledge and awareness about antibiotics and AMR in Vietnam.

**Methods:**

A cross-sectional survey was conducted online in December 2021, featuring 20 questions on antibiotics, AMR, and participants’ habits, attitudes, and potential solutions. The survey was distributed via social media platforms such as Facebook, Zalo, Viber, and WhatsApp. The target sample included Vietnamese working adults above 18 years old. Responses were coded and analyzed using SPSS version 21 and Microsoft Excel version 16.5. Participants were categorized into high, intermediate, and low knowledge levels based on their scores (>80%, 51–79%, and <50%).

**Results:**

A total of 866 Vietnamese adults participated. Most participants (90%) had moderate to high knowledge of antibiotics and AMR. However, only 32.8% knew that 75% of antibiotics are used in agriculture. Knowledge levels varied significantly across demographics such as gender, age, education, profession, and antibiotic use history. Healthcare-related professionals had significantly higher knowledge of antibiotics and AMR than nonhealthcare professionals (*p* < 0.001). Those with health-focused educational backgrounds also had higher knowledge levels (*p* < 0.001). Despite being aware that it was inappropriate, many participants reported discontinuing antibiotics before completing the course prescribed by their doctors.

**Conclusions:**

Age, education, profession, and antibiotic use history positively influenced AMR knowledge. However, even among health-related fields, understanding was only moderate. This indicates a need for enhanced public education to improve knowledge and attitudes toward antibiotics and AMR.

## Introduction

Antimicrobial resistance (AMR) poses significant public health challenges, including longer hospital stays, higher mortality rates, and substantial economic burdens, all leading to intangible costs. In 2007, AMR increased hospitalizations by 2.5 million and caused 25,000 annual deaths in European hospitals, costing about 1.5 billion Euros.^1,2^ By 2015, the situation had worsened considerably, with an estimated 671,689 infections caused by antibiotic-resistant bacteria and approximately 33,110 attributable deaths in the EU/EEA,^3^ reaching an estimated €1.1 billion annually.^4^ In the United States, AMR accounted for 23,000 deaths annually, with direct costs exceeding 20 billion USD and indirect costs over 30 billion USD per year.^5^

Vietnam faces a high rate of drug-resistant bacteria, with hospitals in the south reporting multidrug resistance rates of 74.6% for *E. coli*, nearly 60% for *K. pneumoniae*, and 90% for *A. baumannii.* ^6,7^ Resistance to carbapenem, a “Watch” antibiotic class in the WHO Aware list, has reached 50%, especially in gram-negative bacteria carrying beta-lactamase genes.^7^ From 2009 to 2018, antibiotic sales doubled, with 88% of urban and 91% of rural antibiotics sold without prescriptions.^8,9^

Recognizing the global threat of AMR, the World Health Assembly adopted an action plan in 2015 emphasizing public education and training on AMR.^10^ Since 2013, Vietnam’s Ministry of Health has also implemented a NAP addressing public health policies, education, professional training, research, and collaboration.^11,12^ Inappropriate antibiotic use remains the main driver of AMR, influenced by educational level, profession, and age.^13–16^ This study assessed public awareness and knowledge of antibiotic use and AMR in Vietnam, examining demographic factors to identify knowledge gaps and inform targeted campaigns and policies tackling this issue.

## Methods

### Study design and participants

An online anonymous cross-sectional survey was conducted from December 3, 2021, to December 15, 2021, via Google Forms to assess public knowledge of antibiotic use and AMR in Vietnam. Participants were recruited via social media platforms such as Facebook, Zalo, Viber, and WhatsApp, through university networks, healthcare groups, and personal connections, targeting all individuals aged 18+ who consented to participate. Respondents were grouped by profession into health and nonhealth workers.

### Questionnaire construction

The questionnaire (Appendices, Table A1), adapted from World Health Organization (WHO), was initially tested on a small group of volunteers for feedback and validation before being widely distributed. It assessed knowledge of antibiotics, AMR, deliberate behaviors, and opinions on AMR awareness campaigns and programs.

### Data analysis

Responses were coded and analyzed using SPSS 21 and Microsoft Excel 16.5. Demographics were analyzed with descriptive statistics, presented as frequencies and percentages. Females and ages 18–30 were reference categories for gender and age, while health workers were reference categories for education and profession.

The association between knowledge levels about antibiotics and AMR and demographic factors—including gender, age, educational background, profession, hospitalization history, antibiotic use history, and attitudes toward antibiotics—was analyzed using the *χ* ^2^ test in STATA16. The strength of these associations was evaluated using Cramer’s *V*.

## Results

### General characteristics of respondents

A total of 866 respondents participated, with 298 (65.6%) males and 568 (34.4%) females (Table 1). They were distributed within five age groups 18–30 years (507), 31–40 years (173), 41–50 (128), and above 50 (58). In addition, 372 (43.0%) respondents had a health educational background (Veterinary Medicine—Medicine—Pharmacy); while 53.5% of total adults worked in the health sector.

**Table 1.** General statistics of respondents

		*N* (%)
**Gender**	Female	568 (65.6)
	Male	298 (34.4)
**Age**	18–30	507 (58.5)
	31–40	173 (20.0)
	41–50	128 (14.8)
	51–60	45 (5.2)
	Above 60	13 (1.5)
**Educational background**	Social science	197 (22.7)
	Natural Science—Technology—Engineering (except Biology)	134 (15.5)
	Biology/Biotechnology	163 (18.8)
	Veterinary Medicine—Medicine—Pharmacy	372 (43.0)
**Career (*n* = 482)**	Social science	141 (29.3)
	Natural Science—Technology—Engineering (except Biology)	52 (10.8)
	Biology/Biotechnology	31 (6.4)
	Veterinary Medicine—Medicine—Pharmacy	258 (53.5)
**Hospitalization within the last 6 months**	Yes	47 (5.4)
	No	819 (94.6)
**Antibiotics usage**	Currently using	50 (5.8)
	Within 6 months	251 (29.0)
	More than 6 months ago	377 (43.5)
	Never	31 (3.6)
	Cannot remember	157 (18.1)
**Source of antibiotic (last use)**	Hospital, pharmacy, with prescriptions	539 (62.2)
	Pharmacy, without prescription	236 (27.3)
	Reuse leftover antibiotics	20 (2.3)
	Online (Shopee, Lazada, Facebook, and so on), acquaintances	2 (0.2)
	Never used antibiotics	25 (2.9)
	Do not remember	44 (5.1)

### Antibiotic consumption habits and knowledge of antibiotics

**Figure 1.**
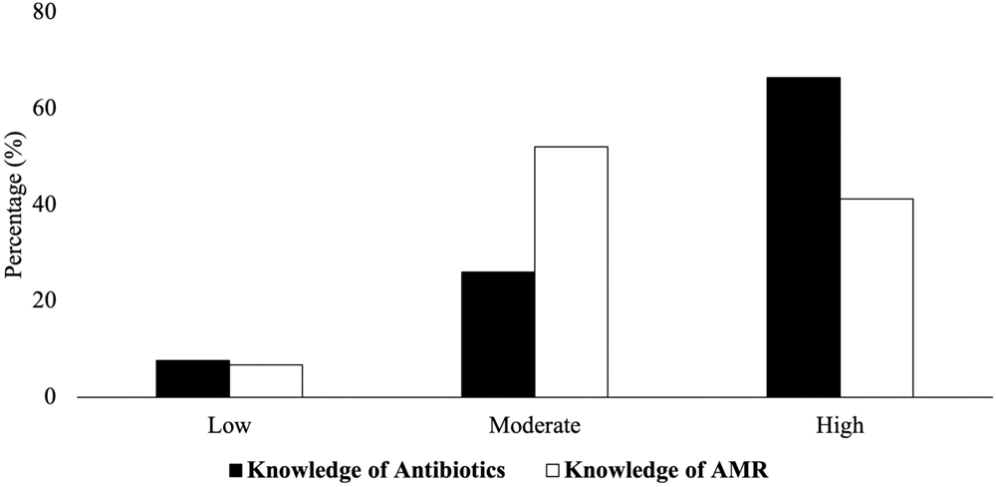
Antibiotic consumption habits reported by participants.

Habits of antimicrobial use were assessed with four statements (Figure 1; Appendices, Table A2). While over 80% of respondents reported reading instructions and avoiding antibiotics for colds or fevers, 22.1% reused leftover antibiotics, and 40% stopped taking them when feeling better. This indicates that understanding proper antibiotic use does not always translate to good consumption habits.

**Figure 2.**
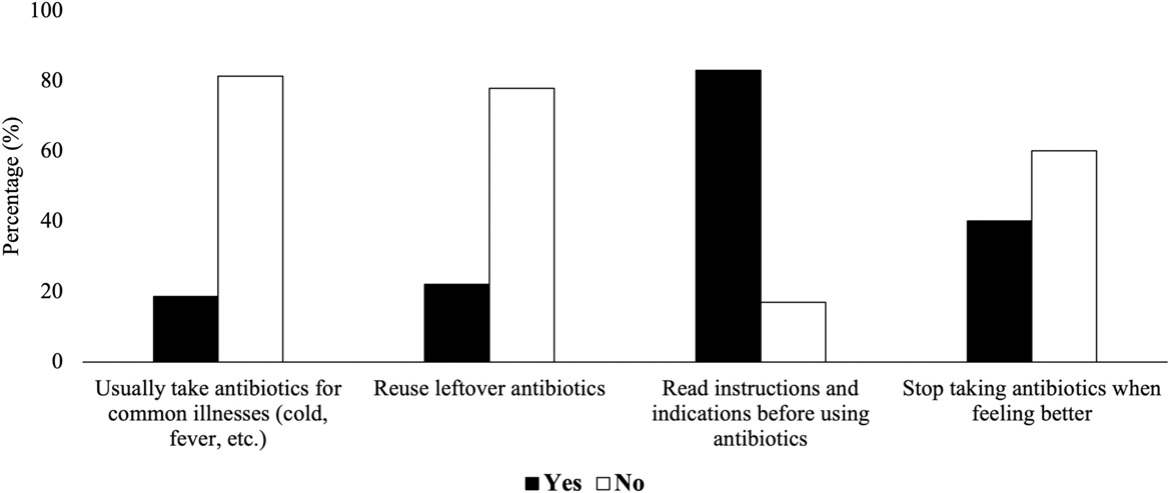
Knowledge of antibiotics and AMR in participants. Knowledge scores of participants were calculated and classified as high, moderate, and low based on the number of accurate responses to statements in the survey.

When quizzed on antibiotics, most respondents demonstrated a general understanding of their purpose, effects, common types, and correct usage. Over 80% answered all ten knowledge questions correctly (Figure 2; Appendices, Table A3–4). Of these, 66.4% had high knowledge (≥80% correct), and 25.98% showed moderate knowledge (≥50% correct). However, only 32.9% were aware that 75% of antibiotics are used in agriculture (Appendices, Table A3).

### Knowledge and attitudes about AMR

The analysis of participants’ knowledge and attitudes toward AMR is summarized in Figure 2, Table 2, and Appendices, Table A3–4. While 95.8% reported familiarity with AMR, most exhibited moderate (52.08%) to high (41.22%) knowledge levels. However, 54.3% incorrectly believed “AMR bacteria require a higher dose of antibiotics to treat” (Appendices, Table A3). Regarding attitudes, 17.0% were neutral, and 9.1% agreed with “AMR can be solved soon” (Table 2). Although 84.9% viewed AMR as a public health concern, 27.8% believed it affects only frequent antibiotic users, and a 37.0% held the view that they would not be personally affected if they used antibiotics appropriately.

**Table 2.** Attitude about the AMR problem—perspective on the scope of the AMR problem

	Agree *N* (%)	Disagree *N* (%)	Neither agree/ disagree *N* (%)
The agriculture sector needs to reduce the overuse of antibiotics	732 (84.5%)	17 (2.0%)	117 (13.5%)
AMR can affect me and my family	798 (92.1%)	22 (2.5%)	46 (5.3%)
AMR is a problem in all countries, except Vietnam	84 (9.7%)	735 (84.9%)	47 (5.4%)
I do not need to worry about antibiotics resistance, it can be solved soon	79 (9.1%)	640 (73.9%)	147 (17.0%)
If I use antibiotics correctly, I will never get infected with antibiotic-resistant bacteria	320 (37.0%)	395 (45.6%)	151 (17.4%)

### Demographic factors associated with knowledge of antibiotics and AMR

Antibiotic knowledge levels varied significantly by gender, age, education, profession, and antibiotic use history but not by hospitalization history (Appendices, Table A5). Male participants showed greater disparities, with higher percentages in both “high” and “low” knowledge levels. Participants aged 31+ had significantly more knowledge than younger groups, while those with health-related backgrounds or prior antibiotic use demonstrated notably higher knowledge (Cramer’s *V* > 0.25).

AMR knowledge was significantly influenced by age, education, profession, and antibiotic use history (Appendices, Table A6). The proportion of “high-level” AMR knowledge decreased with age, from the highest in the 18–30 group to the lowest in those aged 50+. Participants with health-related backgrounds or prior antibiotic use had better AMR knowledge than others. The history of antibiotic use also affects the level of AMR knowledge in which people who used to use antibiotics have a higher level of AMR understanding than those who have never used it.

### Opinions about antibiotics and AMR awareness promotion

**Figure 3.**
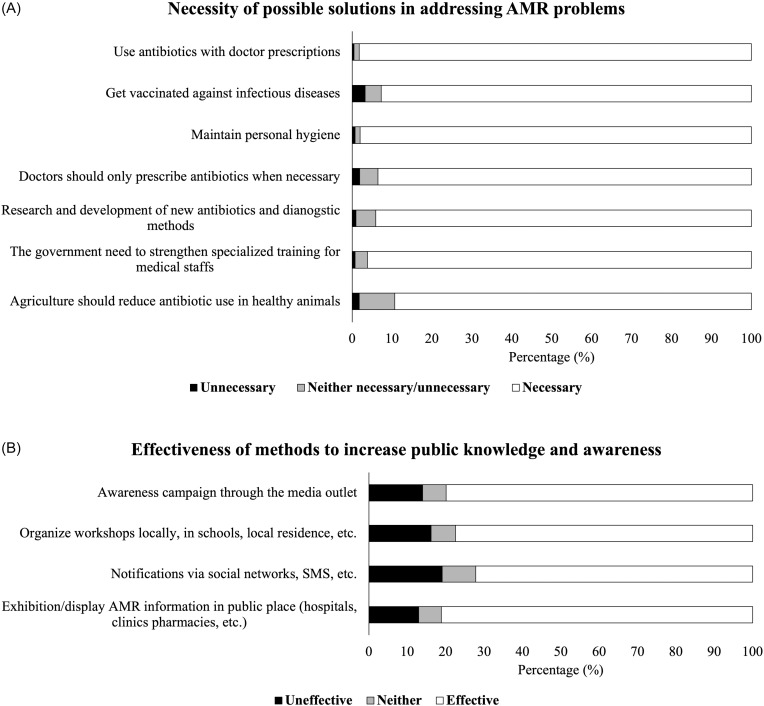
Public opinions on (A) potential solutions to the AMR problems and (B) the effectiveness of different outreach methods in improving public knowledge about antibiotics and AMR.

The majority of respondents agree about the necessity of the suggested AMR fighting measures (Figure 3; Appendices, Table A9–10). The least agreed-upon solutions were in reducing antibiotic use in agriculture (89.38%) and keeping vaccination up-to-date (92.73%). However, 3.23% and 1.73% of respondents thought that vaccination and reducing antibiotic use in agriculture are not necessary measures in addressing the AMR problem. Generally, there is no significant difference in opinions between demographic factors (age, gender, education, and professions) (data not shown).

Among four outreach options, displaying information in public spaces (hospitals, pharmacies, and so on) was deemed effective by 81.18% of respondents, while only 72.17% thought messages through social media and SMS as effective.

## Discussion

### The general state of antibiotics practice in Vietnam

The emergence of AMR has been a major challenge to public health, and it is linked to the overuse and overprescription of antibiotics in medicine and agriculture. To overcome the AMR problem, cultural contexts such as the prescription and use of antibiotics are of particular interest. The result of the survey demonstrated that approximately 80.0% of respondents had taken antibiotics, 29.0% were within the last 6 months, many of which were without consulting with a doctor. This high prevalence of antibiotic use is of importance, showing, first, the extensive usage of antibiotics and, second, the extensive consequence of AMR, affecting a large proportion of the population.

Previous research suggests that the overprescription and dispensing of antibiotics in Low- and Middle-Income Countries (LMICs) often arise from a lack of comprehension regarding AMR and appropriate antibiotic use among prescribers and dispensers.^17^ Nevertheless, despite widespread awareness and knowledge of AMR, there is still a tendency to misuse antibiotics for treating conditions such as colds, fevers, and skin infections.^18^ Furthermore, it is now very easy for people to purchase antibiotics due to the high density of retail pharmacies in Vietnam, with an average of 102 outlets per 100,000 population. This is significantly higher than the global average of 25 outlets per 100,000 population.^19^ This behavior might be influenced by systemic and sociocultural factors. The healthcare system often faces overcrowding and complex procedures, prompting many individuals to bypass clinical visits in favor of faster access through pharmacies. Moreover, cultural perceptions play a role: professional medical care is often sought only for severe conditions, while minor illnesses are typically managed with over-the-counter medications based on prior experience or informal advice from family members. In Vietnam, pharmacy personnel exhibit a diverse understanding of antibiotics and AMR, however, they frequently resort to prescribing antibiotics to patients in pursuit of rapid treatment outcomes. Previous studies highlight a notable disparity between pharmacists’ theoretical knowledge and its practical application.^20,21^ Moreover, certain Vietnamese communities, particularly those in rural areas, lack sufficient knowledge about antibiotics and awareness of AMR.

Considering the current regulations and practices where antibiotics are widely available in pharmacies and can be obtained without a doctor’s prescription, the above-average high prevalence of antibiotics usage, the apparent misuse of antibiotics, and the lack of awareness among the population are all alarming.

### Public awareness of antibiotics and AMR: national and global perspectives

In this study, public knowledge about antibiotics and AMR was found to correlate with the education/professions of the respondents, gender, age, and history of antibiotic use. Understandably, knowledge tends to increase with age and the frequency of antibiotic use, or it is associated well with education level. In a regional context, Ha et al. found that in the northern highlands of Vietnam, only 55.8% of respondents were aware of AMR.^22^ This difference may come from the slight bias of the studied population. In our study, the survey was shared online via the network of our research team who are based in universities, medical centers, and hospitals and mostly in the south of Vietnam. Thus, it can influence the study population and increase awareness of antibiotics compared to the general population.

Education and income levels were again shown to be strong predictors of AMR knowledge. Nguyen et al. found that 86.8% of 1,626 participants purchased antibiotics without a prescription, demonstrated a reduced understanding of how to use them, and lacked awareness of what AMR entails.^23^ Many of those were of a lower educational level, mostly male, and many were freelance workers. Similarly, Di KN et al. reported that respondents with an educational level above high school had 2.663 times higher knowledge scores than those with lower educational levels.^24^ Higher-income individuals also showed more knowledge about antibiotics and AMR than lower-income individuals.^24^

At the global level, these findings are consistent with patterns observed elsewhere. Muflih et al. found that, in Jordan, people living in urban areas had better knowledge of antibiotics than in rural areas,^16^ while another study found respondents in affluent areas had a better understanding of AMR than those in deprived areas of London.^25^

However, as in our study, a common concern across countries is the persistent gap between knowledge and behavior. While 89.0% knew they should follow through with their antibiotic prescription, 40.0% stopped taking antibiotics when they felt better. This gap between awareness and practice was noted in other studies, specifically among the students of the School of Medicine in Italy, more than 15.0% of the students stopped taking antibiotics when symptoms improved and used leftover antibiotics without consulting a doctor.^15^

In addition, misconceptions remain widespread, especially regarding the role of agriculture in AMR. In our study, 15% did not believe antibiotic regulation in agriculture is necessary, and over 35% believed AMR only affects those who frequently use antibiotics and 37.0% thought that they would not be personally affected if they used antibiotics appropriately. As Widayati, A. et al. reported, the public held inaccurate beliefs about the appropriate usage of antibiotics, these misconceptions might also contribute to the inappropriate use of antibiotics.^26^

### Public health interventions and solutions

Vietnam has been reported to have one of the highest rates of AMR in Asia.^27^ The primary cause might stem from the overconsumption of antibiotics not only in medicine but also in agriculture contributing to the escalating issue of antimicrobial-resistant bacteria in food.^28,29^

Vietnam has made significant policy and institutional progress in addressing AMR over the past two decades. Following the 2010 Hanoi Declaration, Vietnam officially adopted the One Health approach to optimize the health of people, animals, and the environment, especially focusing on the control of zoonotic diseases and AMR.^30,31^ The approach seeks to educate and provide training to young community members through funded fellowships and training opportunities. Mitchell et al. provide evidence for the benefits of a multisectoral approach, including the benefit that this approach increases communication between parties and helps to standardize the solutions and methods for all parties involved.^32^ Our study reinforces this perspective, underscoring that education is among the most critical components for combating AMR and should remain central to future policy development.

This commitment materialized further in 2013 when Vietnam became one of the first countries in WHO’s Western Pacific region to implement a National Action Plan (NAP) on AMR, targeting the human health, livestock, and aquaculture sectors.^11,12,30^ Since then, several concrete advancements have been implemented. In the period 2016–2020, the MoH, in collaboration with the Ministry of Agriculture and Rural Development and Ministry of Natural Resources and Environment, established a national AMR surveillance system (Decision 6211/QD-BYT) and issued several regulatory circulars to guide antibiotic use across hospitals, veterinary medicine, livestock feed, and veterinary prescription.^30^ Academic and research institutions have also played a significant role in supporting the research and management of emerging infectious diseases and antimicrobial-resistant bacteria.

Nonetheless, despite these institutional structures and regulatory advancements, critical challenges remain. The knowledge-practice gap observed in our study, such as widespread self-medication and early discontinuation of antibiotics, highlights the need for a renewed and strengthened national strategy. Future action plans should prioritize sustained enforcement, public education, and local-level engagement to ensure a broader and more equitable impact.

### Strategic action plan to enhance an understanding of antibiotics and AMR in the general public

AMR has risen across medicine and agriculture, partly due to the widespread use of antibiotics in livestock production. Food and food-related products can also serve as vectors for antimicrobial-resistant bacteria, contributing to the spread of AMR. Urgent action is needed to boost awareness and responsible antibiotic use. This study proposes strategies for surveillance, education, and communication at national and international levels to combat AMR.

**Figure 4.**
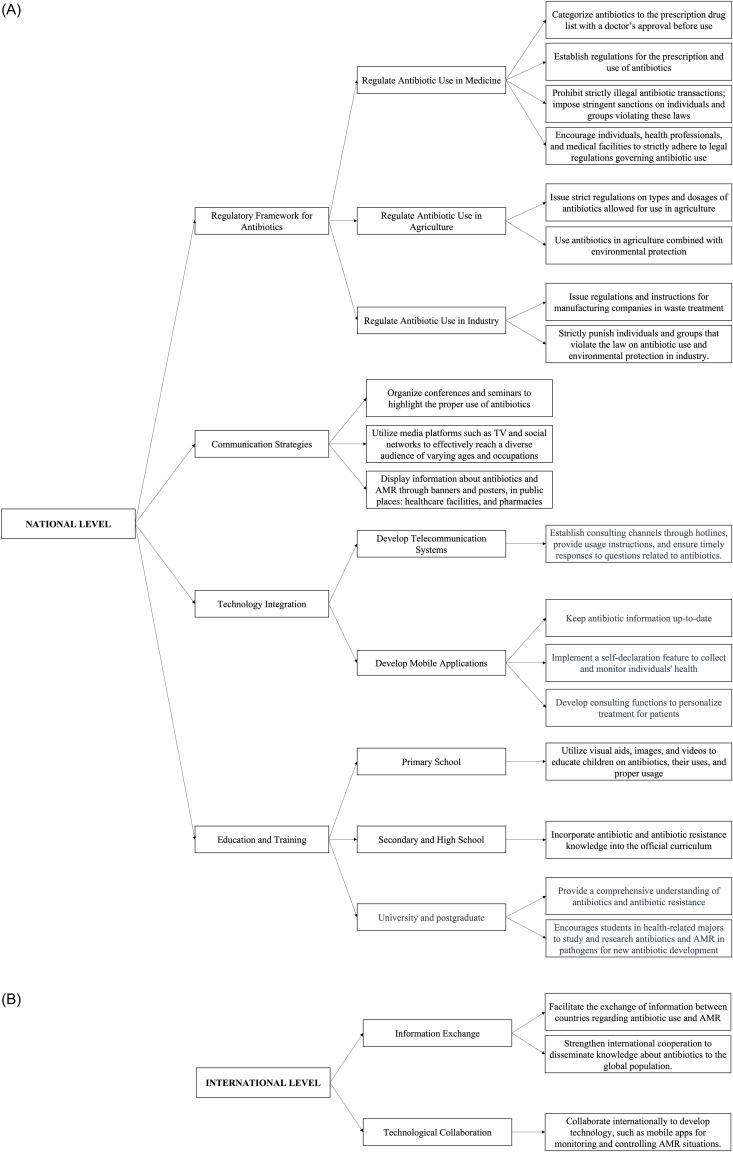
Strategic action plans at (A) national and (B) international levels to to improve public knowledge, attitudes, and awareness regarding antibiotic use and AMR.

Nationally, legislation regulating antibiotic use in healthcare, agriculture, and industry is critical. Collaboration between the MoH, Ministry of Agriculture, and Ministry of Environment is essential to establish rules on antibiotic acquisition, usage, and disposal, with regular inspections ensuring compliance. Effective communication strategies targeting all citizens, especially in remote areas, should leverage mass media and smart technologies, supported by government funding. Tailored educational curricula are vital to fostering responsible antibiotic use (Figure 4).

Globally, combating AMR requires international cooperation in information sharing and support. Organizations like WHO and FAO play crucial roles in connecting nations and aiding underdeveloped regions (Figure 4).

### Strengths and limitations

This study’s strength lies in its large respondent pool, enabling a robust and up-to-date evaluation of public knowledge on antibiotic use and AMR. The findings reveal critical gaps between awareness and behavior, offering valuable insights for developing national strategies to enhance public knowledge and reduce AMR-related healthcare burdens. However, the study has limitations. (1) The online convenience sampling may have introduced nonresponse bias, with younger, female, and health-sector-employed individuals being overrepresented, potentially skewing results. (2) Reliance on multiple-choice questions may have encouraged socially desirable responses, obscuring underlying misconceptions. Incorporating qualitative methods could provide deeper insights into these misconceptions and respondent perspectives. (3) The survey was conducted briefly during the period of the COVID-19 pandemic, which potentially influenced the results due to increased public awareness of infectious diseases, including AMR. Therefore, similar studies should be conducted under different time frames and social contexts to assess the robustness and generalizability of the findings.

## Conclusion

This study explored public awareness and attitudes toward antibiotics and AMR in Vietnam. While most respondents were aware of antibiotics and AMR, this knowledge often failed to translate into proper antibiotic use. Awareness levels varied by age and health-sector education, emphasizing the need for targeted educational interventions to correct misconceptions and promote responsible behaviors.

## Supporting information

10.1017/ash.2025.10034.sm001 Tran et al. supplementary material Tran et al. supplementary material

## Supplementary material

The supplementary material for this article can be found at https://doi.org/10.1017/ash.2025.10034.
