# Supplementary material for: Public knowledge and attitudes towards antibiotics and antimicrobial resistance (AMR) in vietnam: a cross-sectional study
Source: Antimicrob Steward Healthc Epidemiol. 2025 Jul 31;5(1):e165. doi: 10.1017/ash.2025.10034 (PMC12322788; doi:10.1017/ash.2025.10034)
Supplement: Tran et al. supplementary material [file S2732494X2510034Xsup001.docx]

**SUPPLEMENTARY INFORMATION**

**Table A1. Questionaire.**

| **Questions** | **Options** |
| --- | --- |
| **I. GENERAL INFORMATION** | |
| **1. Gender?** | Female |
|  | Male |
| **2. How old are you?** | 18-30 |
|  | 31-40 |
|  | 41-50 |
|  | 51-60 |
|  | Above 60 |
| **3. Where do you live?** |  |
| **4. You are…** | Undergraduate student |
|  | MSc student |
|  | PhD candidate |
|  | Others |
| **5. If you are student, what is your major?** | Social science |
|  | Natural Science - Technology - Engineering (except Biology) |
|  | Biology/Biotechnology |
|  | Veterinary Medicine - Medicine - Pharmacy |
| **6. If you are worker, what is your educational background?** | Social science |
|  | Natural Science - Technology - Engineering (except Biology) |
|  | Biology/Biotechnology |
|  | Veterinary Medicine - Medicine - Pharmacy |
| **7. If you are worker, what is your occupation?** | Social science |
|  | Natural Science - Technology - Engineering (except Biology) |
|  | Biology/Biotechnology |
|  | Veterinary Medicine - Medicine - Pharmacy |
| **8. In the last 6 months, have you ever been hospitalized?** | Yes |
|  | No |
| **9. Have you taken any antibiotics in the last 6 months?** | Currently using |
|  | Within 6 months |
|  | More than 6 months ago |
|  | Never |
|  | Cannot remember |
| **10. Where did you get antibiotics in the last taking it?** | Hospital, pharmacy, with prescriptions |
|  | Pharmacy, without prescription |
|  | Reuse leftover antibiotics |
|  | Online (shopee, lazada, facebook,...), acquaintances |
|  | Never used antibiotics |
|  | Do not remember |
| **II. HABITS WHEN USING ANTIBIOTICS** | |
| **1. Do you have any of following habitats when using antibiotics? (Yes/No)** | Usually take antibiotic for common illness (cold, fever, etc.) |
|  | Reuse leftover antibiotics |
|  | Read instruction and indications |
|  | Stop taking antibiotic when feeling better |
| **2. Where do you get information about antibiotics?** |  |
| **III. KNOWLEDGE OF ANTIBIOTICS** | |
| **1. Do you think the following statements are true or false about antibiotics? (True/False)** | Antibiotic is used to treat bacterial infections |
|  | Antibiotic is used to treat viral infections |
|  | Antibiotic can kill beneficial bacteria in the body |
|  | Antibiotic does not cause any side effects |
|  | Paracetamol is an antibiotic |
|  | Aspirin is an antibiotic |
|  | Penicilin is an antibiotic |
| **2. Is antibiotics used in the following case true or false? (True/False)** | We can use the same antibiotic as our family and friends, as long as it treats the same illness |
|  | When the symptoms aleviate, we should stop using antibiotic immediately |
|  | Only use antibiotic according to doctor prescription |
| **3. Did you know that 75% of all antibiotics in Vietnam are used in agriculture?** | Yes |
|  | No |
| **IV. KNOWLEDGE ABOUT AMR** | |
| **1. Have you ever heard about AMR?** | Yes |
|  | No |
| **2. Do you think the following statements are true or false about AMR? (True/False)** | Antibiotics resistant bacteria need higher dose of antibiotic to treat |
|  | The infection of bacteria become more serious when it resistant to antibiotics |
|  | Overuse of antibiotics can lead to AMR |
|  | AMR only occurs in people who regularly use antibiotics |
|  | Infection with antibiotic-resistant bacteria can increase the risk in surgery, organ transplants, cancer therapy. |
| **3. On the scale below, do you agree or disagree with the following statements? (Agree/Disagree/No idea)** | The agriculture sector needs to reduce the overuse of antibiotics |
|  | AMR can affect me and my family |
|  | AMR is a problem in all countries, except Vietnam |
|  | I do not need to worry about AMR, it can be solve soon |
|  | If I use antibiotic correctly, I will never get infected with AMR bacteria |
| **V. OPINIONS ABOUT ANTIBIOTICS AND AMR AWARENESS PROMOTION** | |
| **1. Assess the necessary of following ways to prevent AMR (Unnecessary/Neither/Necessary)** | Use antibiotic under doctor's íntruction |
|  | Get vaccinated against infectious diseases |
|  | Maintain personal hygiene |
|  | Doctors should only prescribe antibiotics to patients when it necessary |
|  | Research and development of new antibiotics and diagnostic methods |
|  | The government need to strengthen specialized training for medical staff |
|  | Agriculture should reduce antibiotic use in healthy animals |
| **2. Evaluate effectiveness of strategies to promote AMR in community (Uneffective/Neither/Effective)** | Dissemination of knowledge through the media |
|  | Organize worskshop in school,... |
|  | Notifications via social networks, SMS... |
|  | Exhibition/display AMR in public place (hospitals, clinics, pharmacies, etc.) |
| Questionaire validation: A pilot study was conducted on 30 randomly chosen respondents. The respondents were asked to fill in the questionnaire and were allowed to comment on the clarity of the questions. Afterward, minor amendments were made according to the comments of the participants. | |

Table A2. Antibiotic consumption habits, N (%)

|  | **Yes**  **N (%)** | **No**  **N (%)** |
| --- | --- | --- |
| Usually take antibiotics for common illnesses (cold, fever, etc.) | 162 (18.7) | 704 (81.3) |
| Reuse leftover antibiotics | 191 (22.1) | 675 (77.9) |
| Read instructions and indications before using antibiotics | 719 (83.0) | 147 (17.0) |
| Stop taking antibiotics when feeling better | 346 (40.0) | 520 (60.0) |

Table A3. Knowledge of antibiotics and AMR in participants.

|  |  |  | **Correct answer** | **Answered correctly (%)** |
| --- | --- | --- | --- | --- |
| **Knowledge of Antibiotics** | **Purpose/effects of antibiotics** | Antibiotics are used to treat bacterial infections | Yes | 89.6% |
|  |  | Antibiotics are used to treat viral infections | No | 77.6% |
|  |  | Antibiotics can kill beneficial bacteria in the body | Yes | 84.8% |
|  |  | Antibiotics do not cause any side effects | No | 94.9% |
|  | **Which drugs are antibiotics** | Paracetamol is an antibiotic | No | 87.4% |
|  |  | Aspirin is an antibiotic | No | 73.9% |
|  |  | Penicillin is an antibiotic | Yes | 93.3% |
|  | **Usage of antibiotics** | We can use the same antibiotics as our family and friends, as long as it treats the same illness | No | 85.3% |
|  |  | When the symptoms alleviate, we should stop using antibiotics immediately | No | 80.8% |
|  |  | Only use antibiotics according to the doctor's prescription | Yes | 95.7% |
|  | **Antibiotics in agriculture** | Over 75% of antibiotics are used in the agricultural sector | Yes | 32.9% |
| **Knowledge of AMR** |  | Antibiotics resistant bacteria need higher doses of antibiotics to treat | Yes | 45.7% |
|  |  | The infection of bacteria becomes harder to treat when it resistant to antibiotics | Yes | 97.8% |
|  |  | Overuse of antibiotics can lead to AMR | Yes | 98.3% |
|  |  | AMR only occurs in people who regularly use antibiotics | No | 72.2% |
|  |  | Infection with antibiotic-resistant bacteria can increase the risk of surgery, organ transplants, and cancer therapy. | Yes | 97.1% |

**Table A4. Levels of antibiotic and AMR knowledge in participants.**

|  | **Knowledge Rank** | | |
| --- | --- | --- | --- |
|  | **Low**  **N (%)** | **Moderate**  **N (%)** | **High**  **N (%)** |
| **Knowledge of Antibiotics** | 66 (7.62) | 225 (25.98) | 575 (66.4) |
| **Knowledge of AMR** | 58 (6.7) | 451 (52.08) | 357 (41.22) |
| Evaluation was based on answers to knowledge of antibiotic and AMR sections. Antibiotic knowledge were evaluated using 10 items; the scores ranged between 0 and 10. Knowledge of AMR was measured by 11 items; the scores ranged between 0 and 11. Those who correctly answered more than 80%, 51-79%, and less than 50% of the total questions were regarded to have a high, intermediate, and low level of knowledge about antibiotics and AMR, respectively. | | | |

Table A5. Factors associated with antibiotics knowledge

| **Factors** | | **N** | **Antibiotic Knowledge rank** | | | **Cramer's V** | **CI 95%** | **p value** |
| --- | --- | --- | --- | --- | --- | --- | --- | --- |
|  |  |  | **Low**  **N (%)** | **Moderate**  **N (%)** | **High**  **N (%)** |  |  |  |
| **Gender** | **Female** | 568 | 39 (6.87) | **162 (28.52)** | 367 (64.61) | 0.0841 | 0.0231157 - 0.1450687 | **0.047** |
|  | **Male** | 298 | 27 (9.06) | **63 (21.14)** | 208 (69.8) |  |  |  |
| **Age** | **18-30** | 507 | **55 (10.85)** | **148 (29.19)** | **304 (59.96)** | 0.1386 | 0.1017674 - 0.1753397 | **<0.001** |
|  | **31-40** | 173 | **7 (4.05)** | 38 (21.97) | **128 (73.99)** |  |  |  |
|  | **41-50** | 128 | **2 (1.56)** | 32 (25) | 94 (73.44) |  |  |  |
|  | **Over 50** | 58 | 2 (3.45) | **7 (12.07)** | **49 (84.48)** |  |  |  |
| **Education Background** | **Health** | 535 | **11 (2.06)** | **80 (14.95)** | **444 (82.99)** | 0.4565 | 0.3966528 - 0.5163023 | **<0.001** |
|  | **Non-health** | 331 | **55 (16.62)** | **145 (43.81)** | **131 (39.58)** |  |  |  |
| **Professional groups** | **Health** | 289 | **5 (1.73)** | **26 (9)** | **258 (89.27)** | 0.4109 | 0.3295925 - 0.4921474 | **<0.001** |
|  | **Non-health** | 193 | **18 (9.33)** | **73 (37.82)** | **102 (52.85)** |  |  |  |
| **Hospitalization** | **No** | 819 | 61 (7.45) | 214 (26.13) | 544 (66.42) | 0.0289 | (-)0.026056 - 0.0838072 | 0.697 |
|  | **Yes** | 47 | 5 (10.64) | 11 (23.4) | 31 (65.96) |  |  |  |
| **Antibiotic use history** | **No** | 188 | **24 (12.77)** | **66 (35.11)** | **98 (52.13)** | 0.1644 | 0.095147 - 0.2336063 | **<0.001** |
|  | **Yes** | 678 | **42 (6.19)** | **159 (23.45)** | **477 (70.35)** |  |  |  |

Adjusted residual greater than 1.96 were highlighted in bold as they are more extreme than what would be expected if the null hypothesis of independence was true.

Table A6. Factors associated with AMR knowledge.

| **Factors** | | **N** | **AMR Knowledge rank** | | | **Cramer's V** | **CI 95%** | **p value** |
| --- | --- | --- | --- | --- | --- | --- | --- | --- |
|  |  |  | **Low**  **N (%)** | **Moderate**  **N (%)** | **High**  **N (%)** |  |  |  |
| **Gender** | **Female** | 568 | 42 (7.39) | 299 (52.64) | 227 (39.96) | 0.0472 | (-)0.0070305 - 0.101499 | 0.381 |
|  | **Male** | 298 | 16 (5.37) | 152 (51.01) | 130 (43.62) |  |  |  |
| **Age** | **18-30** | 507 | **42 (8.28)** | **241 (47.53)** | **224 (44.18)** | 0.1014 | 0.0604564 - 0.1422996 | **0.007** |
|  | **31-40** | 173 | 10 (5.78) | 90 (52.02) | 73 (42.2) |  |  |  |
|  | **41-50** | 128 | 4 (3.13) | **82 (64.06)** | **42 (32.81)** |  |  |  |
|  | **Over 50** | 58 | 2 (3.45) | **38 (65.52)** | 18 (31.03) |  |  |  |
| **Education Background** | **Health** | 535 | **22 (4.11)** | **247 (46.17)** | **266 (49.72)** | 0.2351 | 0.171736 - 0.2984563 | **<0.001** |
|  | **Non-health** | 331 | **36 (10.88)** | **204 (61.63)** | **91 (27.49)** |  |  |  |
| **Professional groups** | **Health** | 289 | 13 (4.5) | **141 (48.79)** | **135 (46.71)** | 0.1507 | 0.0649524 - 0.236442 | **0.004** |
|  | **Non-health** | 193 | 11 (5.7) | **121 (62.69)** | **61 (31.61)** |  |  |  |
| **Hospitalization** | **No** | 819 | 56 (6.84) | 431 (52.63) | 332 (40.54) | 0.0592 | 0.002185 - 0.1162139 | 0.219 |
|  | **Yes** | 47 | 2 (4.26) | 20 (42.55) | 25 (53.19) |  |  |  |
| **Antibiotic use history** | **No** | 188 | **21 (11.17)** | 109 (57.98) | **58 (30.85)** | 0.1318 | 0.0639085 - 0.1997092 | **0.001** |
|  | **Yes** | 678 | **37 (5.46)** | 342 (50.44) | **299 (44.1)** |  |  |  |

Adjusted residual greater than 1.96 were highlighted in bold as they are more extreme than what would be expected if the null hypothesis of independence was true.

**Table A7. Public opinions regarding prospective measures for the prevention of AMR.**

|  | **Unnecessary**  **N (%)** | **Neither**  **N (%)** | **Necessary**  **N (%)** |
| --- | --- | --- | --- |
| Use antibiotics with doctor prescriptions | 4 (0.46) | 11 (1.27) | 851 (98.27) |
| Get vaccinated against infectious diseases | 28 (3.23) | 35 (4.04) | 803 (92.73) |
| Maintain personal hygiene | 6 (0.69) | 11 (1.27) | 849 (98.04) |
| Doctors should only prescribe antibiotics when necessary | 16 (1.85) | 40 (4.62) | 810 (93.53) |
| Research and development of new antibiotics and dianogstic methods | 8 (0.92) | 43 (4.97) | 815 (94.11) |
| The government need to strengthen specialized training for medical staffs | 6 (0.69) | 27 (3.12) | 833 (96.19) |
| Agriculture should reduce antibiotic use in healthy animals | 15 (1.73) | 77 (8.89) | 774 (89.38) |

# Table A8. Public opinions on the efficacy of various outreach approaches in enhancing public awareness regarding antibiotics and AMR.

|  | **Uneffective**  **N (%)** | **Neither**  **N (%)** | **Effective**  **N (%)** |
| --- | --- | --- | --- |
| Awareness campaign through the media outlet | 121 (13.97) | 53 (6.12) | 692 (79.91) |
| Organize workshops locally, in schools, local residence, etc. | 140 (16.17) | 55 (6.35) | 671 (77.48) |
| Notifications via social networks, SMS, etc. | 165 (19.05) | 76 (8.78) | 625 (72.17) |
| Exhibition/display AMR information in public place (hospitals, clinics pharmacies, etc.) | 112 (12.93) | 51 (5.89) | 703 (81.18) |
